# The physical and optical investigations of the tannic acid functionalised Cu-based oxide nanostructures

**DOI:** 10.1038/s41598-022-14281-z

**Published:** 2022-06-14

**Authors:** Nurul Akmal Che Lah, Puhanes Murthy, Mohd Nashrul Mohd Zubir

**Affiliations:** 1 Faculty of Manufacturing & Mechatronics Engineering Technology (FTKPM), Universiti Malaysia Pahang, Pekan, 26600 Pahang, Malaysia; 2 Centre of Excellence for Advanced Research in Fluid Flow (CARiFF), Universiti Malaysia Pahang, Gambang, 26300 Pahang, Malaysia; 3 Department of Mechanical Engineering, Faculty of Engineering, Level 3, Engineering Tower, University of Malaya, 50603 Kuala Lumpur, Malaysia

**Keywords:** Energy science and technology, Engineering, Materials science, Nanoscience and technology, Physics

## Abstract

The need for a mild, low-cost, green environment that is able to produce exotic properties of output nanostructures is appealing nowadays. Employing these requirements, the copper (Cu)—based oxide nanostructures have been successfully synthesised via one-pot reaction using biocompatible natural polyphenol, tannic acid (TA) as both the reducing agent and stabiliser at 60, 70 and 80 °C. The structural and optical studies disclosed the effect of TA on the surface morphology, phase purity, elemental composition, optical microstrain and optical intrinsic energy of this mixed Cu_2_O and CuO nanostructures. The optically based method describes the comparative details of the multi-band gap in the presence of more than one element with overlapping spectra from the first-derivative absorbance curve $\frac{\Delta E}{\Delta A}$ and the exponential absorbance of Urbach tail energy ${E}_{U}$ towards the conventional Tauc bandgap. The $\frac{\Delta E}{\Delta A}$ demonstrates that the pronounced effect of TA that Cu_2_O and CuO nanostructures creates much sensitive first-derivative bandgap output compared to the Tauc bandgap. The results also show that the ${E}_{U}$ reduced as the temperature reaches 70 °C and then experienced sudden increase at 80 °C. The change in the pattern is parallel to the trend observed in the Williamson–Hall microstrain and is evident from the variations of the mean crystallite size ${D}_{m}$ which is also a cause response to the change in temperature or pH. Therefore, the current work has elucidated that the structural and optical correlations on the as-synthesised Cu_2_O and CuO nanostructures in the presence of TA were the combined reaction of pH change and the ligand complexation reactions. The acquired results suggest a more comprehensive range of studies to further understand the extent relationship between the physical and optical properties of TA functionalised Cu-based oxide nanostructures.

## Introduction

Functionalisation of copper (Cu) -based oxide nanostructures has become essential in excitonics industrial device applications that manifest in the conversion and transport of energy such as modern catalytic power generation and flexible or wearable miniaturise electronic products^1–5^. The exploitation of the intrinsic physical features in nano-based oxide materials that are easily synthesised and fabricated are the key concerns in the development route of these materials. The chemical reduction route is the most utilised method to synthesise complex nanostructures-based particles^6–8^. Cu-based oxide nanostructures belong to the oxide metal group with superior functionalities in the conversion of light into other forms of energy such as in photovoltaic devices and solar harvesters^9–12^. The feasibility of these binary oxides (Cu_2_O and CuO) as a *p*-type super capacitor electrode material shows exceptional light-harvesting capability over the whole visible spectrum and able to accumulate nearly all the photon energy at the quantum level. The incorporation of both Cu_2_O and CuO stimulates bi-phase changes in one system that triggers surprising properties. For instance, the recombination of Cu_2_O and CuO gives high stability with remarkable photocurrent density^13–15^ than the bare Cu_2_O or CuO^16^. These are associated with the remarkable changes in nanostructures assembly or distribution, morphology, crystallinity, mean crystallite size, and energetic disorder (band gap absorption).

The most commonly used copper salt precursors such as Cu(II) nitrate trihydrate, Cu(II) chloride dehydrates and Cu(II) sulfate are known for their ecotoxicological towards the living cells^17,18^. The ecotoxicology is mainly due to the active redox of the Cu ions itself that eases the formation of reactive oxygen species of Cu and therefore, can cause aerosol toxicity. For half of a century, the known cause for fundamental toxicity is related to the number of free cupric ions and their concentration amount that change the chemical speciation of the Cu^19–21^. It should be noted that the redox level of cupric ions (Cu^2+^ or Cu^3+^) is different due to the differences in oxidation states^22–24^. The lability of Cu complex species contributes to this condition. All these factors can be reduced with the attachment of inorganic ligands, which can principally reduce the lability and thus lessen the relative affinity of the ligand-nanostructures. The presence of organic molecule could contribute to much larger variations in terms of the diffusion distance that might account from different kinetic of subsequent reactions.

In this case, the use of natural organic tannic acid (TA) surprisingly leads to the one-pot mixed phase of Cu-based oxide nanostructures without adding extra chemical reagents at designated temperatures and concentrations. TA has polydentate ligand, the large polyphenol of hydrolysable tannin commonly found in wines and teas. It possesses a high tendency to attach to various metal ions (high electrostatic stabilisation), especially ferric or cupric ions, to create new stable metal complexes or chelations with diverse material chemical compositions and surface properties^25–28^. From the clinical health point of view, the primary benefit function of the ‘full’ green TA is the capability to neutralise the free radical of which, as a result, warrant an anti-proliferative effect that causes several cancer cell lines^29–31^. This ultimately influence the characteristic of surface charge and further yield the dissolution of metal release^32–34^. It is believed that TA causes the increase of H^+^ concentration (reduce the Cu^2+^ concentration) due to the formation of chelation bonding when in contact with Cu^2+^. At some point, TA induces dispersion rather than aggregation upon strong repulsion and steric hindrance, indirectly controlled by the moieties' pH. After passing through some pH levels, it inclines to dissolute the Cu as an effect of increased H^+^ concentration and the complexation of TA-Cu^2+^. The polydentate ligand of TA tends to mix with the Cu^2+^ through a fast coordination rate to yield linked metal-polyphenol complex such as CuO-TA as the TA contributes more oxygen to the structures. The fast coordination causes the aggregation of the nanostructures^35–38^. Hence, the unique complex process reactions between the TA ligand and Cu ions to form the Cu_2_O and CuO are essential as it becomes easy to predict the parameters that control the reactions and how long the reaction is taken for the specific response.

A systematic study on the oxidation mechanism and the control of reaction temperature of TA functionalised Cu-based oxide nanostructures has not yet been reported significantly. Herein, the present work reported the influence of progeny TA on the pH value on the structural formation of Cu-based oxide nanostructures with different mean crystallite diameters, ${D}_{m}$, phase purity, elemental composition and microstrain of diffraction lines via the Williamson–Hall method at three different reaction temperatures (60, 70 and 80 °C). The work endows the simplest combined reagents with TA role as both reducing and stabilizing agents at low reaction temperatures. The facile method without a sophisticated multistep synthesis process using water as solvent proves a success. The optical resonances from TA remarkably alter the excited state of Cu ions by means of a strong or weak coupling regime between them and this was further analysed using optical intrinsic Urbach energy. Such behavior led to the overall changes in the pH value of the system and the different sizes of produced particles or nanocrystallisation modulated the microstrain and band gap. The possible correlation between physical and optical properties shows that the core control may initiate from the organisation/dispersion nature of the particulate Cu-based oxide nanostructures.

## Materials and methods

### Materials

All materials were commercially available and used without any additional purification. Copper (II) sulfate (CuSO_4_, anhydrous, powder, ≥ 99.99% trace metals basis) as a source of the precursor was purchased from Sigma Aldrich (Malaysia). Tannic acid (TA, C_76_H_52_O_46_, ACS reagent) as the reducing agent and stabiliser was obtained from Alfa Aeser (Malaysia), respectively. Double distilled water was used as a solvent.

### Synthesis of TA functionalised Cu-based oxide nanostructures

**Figure 1:**
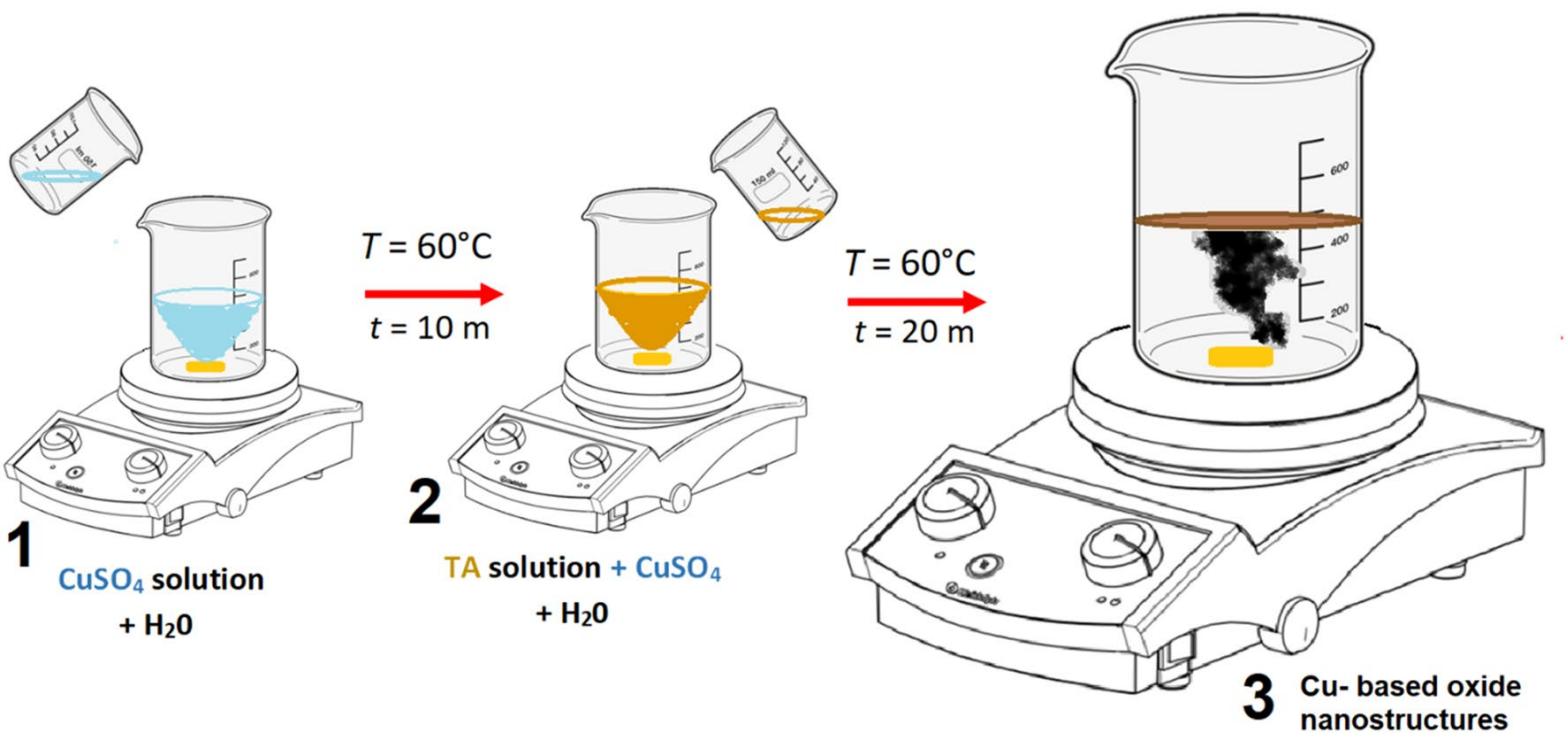
Schematic illustration of the experimental setup for chemical reduction process of Cu-based oxide-TA nanostructures.

The Cu-based oxide-TA nanostructures were synthesised using the chemical reduction method. TA (0.9 g) was mixed with 20 ml of distilled water in a small beaker. CuSO_4_ (1.507 g) was also diluted in 20 ml of double distilled water so that the solution of dissolve salt is ready upon the mixing process to take place. At the same time, 100 ml distilled water was heated to 60 °C. Later, the CuSO_4_ solution was added to the heated distilled water and stirred vigorously for 10 min. The color of the solution changes from colorless to light blue. The color turns from light blue to light brown solutions upon adding TA solution into the mixture. After 20 min of reaction time, the solution color changed to a dark brown solution. The schematic illustration of the one-pot synthesis method used in producing Cu-based oxide nanostructures is demonstrated in Fig. 1. Upon completion of the reaction, the sample was left to cool at room temperature. The sample was then centrifuged to remove any impurities from the solution and washed several times with distilled water and ethanol. Finally, the clean and pure suspension of Cu-based oxide-TA nanostructures was obtained. The method is repeated for temperatures at 70 and 80 °C for comparison.

### pH measurement

The as-synthesised samples at 60, 70 and 80 °C were diluted for 30 µM in 10 ml distilled water for all spectroscopic measurements. The pH measurement was conducted using the pH strip (Natural Care, Park City, UT). The pHs before and after the dilution are shown in Table 1.

**Table 1:** The solution pH initial and after diluted with distilled water for the spectroscopic measurements of 30 µM solution concentration.

	60 °C	70 °C	80 °C
Initial pH	7	7	11
50% diluted pH	7	7	11

### Morphological measurement

The surface morphology of the prepared Cu-based oxide nanostructures was observed using a Field Emission Scanning Electron Microscopy (FESEM, JEOL JSM-7800F, JEOL, Japan) set at an accelerating voltage of 5 kV. Elemental analysis was performed using dispersive energy X-ray (EDX LSM880-FESEM, Carl Zeiss AG, Germany). The typical magnification of FESEM-EDX is in the range of × 50,000 to 100,000. For FESEM-EDX, the solutions were dropped onto the aluminium foil substrate and let dry for 12 h prior to the observation. Also, a 300 kV Transmission Electron Microscope (TEM, 3000F, JEOL, Japan) and equipped with a field emission gun was used for the morphological and size distribution investigations. Samples for TEM were prepared by dropping the suspension of the Cu-based oxide nanostructure onto a lacey carbon-coated grid specimen holder and dried under ambient conditions for 12 h. The TEM micrographs were obtained at different magnifications and further examined using Gatan Micrograph software.

### Crystallisation measurement

The crystal structure of the samples was characterised using X‐ray diffraction (XRD) powder patterns (Bruker D8 Model 2016, Berlin, Germany). The measurement was set under Cu Kα radiation (λ = 0.154 nm) with a scanning range of 10–100 nm at 40 kV and 40 mA in a powder diffraction arrangement. Also, the as equipped dimensional detector was used to scan the crystal phases of the samples. The full-width at half maximum (FWHM) of all samples was obtained for comparative studies on the effect of the crystallite size from the XRD patterns. The dense powder of the sample was prepared by drying the dropped solutions of suspension on the thin glass substrate. The sample was then left to dry in a vacuum cabinet for 12 h.

The unit cell lattice constants *a*, *b* and *c* of CuO and Cu_2_O nanostructures were calculated by using the following monoclinic and cubic lattices equations^39^, respectively:

1 $$\frac{1}{{d}_{hkl}^{2}}=\frac{1}{{\mathrm{sin}}^{2}\beta }\left(\frac{{h}^{2}}{{a}^{2}}+\frac{{{k}^{2}\mathrm{sin}}^{2}\beta }{{b}^{2}}+\frac{{l}^{2}}{{c}^{2}}-\frac{2hl \mathrm{cos}\beta }{ac}\right),$$

2 $$\frac{1}{{d}_{hkl}^{2}}=\frac{({h}^{2}+{k}^{2}+{l}^{2})}{{a}^{2}},$$

with

3 $${d}_{hkl}=\frac{n\lambda }{2 \mathrm{sin}\theta },$$

where λ refers to the wavelength of the incident X-ray beam, *n* corresponds to the order of diffraction (for first-order *n* = 1), ${d}_{hkl}$ is the inter-planar spacing referring to the distance between the parallel planes of atoms and *hkl* is the Miller indices.

The mean crystallite size, ${D}_{m}$ of the as-produced samples for comparing the size structure was calculated using FWHM of all peaks via the Scherer equation^40,41^. Based on the Scherrer equation, ${D}_{m}$ (in nm) is inversely correlated to the peak width β (FWHM) via the following relationship:

4 $${D}_{m}=\frac{0.9\lambda }{\beta \mathrm{cos}\theta }.$$

where λ = 0.1564 nm refers to the wavelength of the X-ray using the CuKα monochromatic radiation, while β and θ are the FWHM of the XRD sample peaks in radians and the Bragg’s angle of the diffraction peak, respectively.

The crystallinity percentage of the sample is calculated from the area of XRD peaks using the relationship of^42–44^:

5 $$Crystallinity=\frac{Area\, of\, crystalline\, peak}{Area\, of\, all \,peaks\, (crystalline+amorphous)}\,\times 100.$$

Meanwhile, the microstrain, $\varepsilon$ is calculated via the Williamson–Hall method using the following equation^45,46^:

6 $${\beta }_{T}\mathrm{cos}\theta =\varepsilon \left(4\sin\theta \right)+K\lambda /{D}_{m},$$

with ${\beta }_{T}={\beta }_{crystallite}+{\beta }_{microstrain}$ in radians, $\theta$ is the diffraction angle position in radians unit, $K=$ 0.9 refers to the shape factor^47–51^, $\lambda = 0.15406$ nm denotes the wavelength of the XRD and ${D}_{m}$ is the average crystallite size.

### Optical measurement

The surface plasmon resonance spectra for the samples were carried out using a UV–Visible (UV–Vis) spectrophotometer (UVS-2700, Labomed Inc., USA). The suspension solution was diluted based on the suspension to a solvent dilution ratio of 1:6. The 4.0 ml diluted suspension was then pipetted into the quartz cell for the test. Prior to the test, a baseline measurement was conducted to measure a 0% correction for each wavelength in the scan to ensure accurate absorbance measurement. The range of absorbance spectra is between 200 and 800 nm.

In the present study, the first-order derivative of energy, $\Delta E$ with respect to the absorbance, $\Delta A$ is calculated and given by^52,53^:

7 $$\frac{\Delta E}{\Delta A}={f}^{^{\prime}}\left(E\right).$$

The optical band gap (${E}_{g}$) of the studied suspension from the high absorption region was calculated by using Tauc relation at room temperature which yields the direct equation for Cu-based oxide nanostructures^54,55^:

8 $$(\alpha h\upsilon {)}^{2}=A\left(h\upsilon -{E}_{g}\right),$$

where *A* is known as the Tauc parameter, which signifies the degree of disorder in the materials and measures the transition probability, $\upsilon$ is the frequency of the incident beam light radiation with $h\upsilon$ the photon energy, $\alpha$ is the absorption coefficient and ${E}_{g}$ is the optical band gap energy. Principally, as for the Tauc, the indirect bandgap specifies the amorphous substances, whereas the direct allowed bandgap from crystalline substances.

In general, the absorption coefficient (α) often displays an exponential tail below the optical bands. These so-called Urbach tails ${E}_{U}$ is defined using the following equation^56,57^:

9 $$\alpha ={\alpha }_{0}\mathrm{exp}\left(\frac{E}{{E}_{U}}\right),$$

with the $\alpha$ refers to spectral dependence of absorbance coefficient, ${\alpha }_{0}$ is the constant, $E$ is known as $h\nu$ that represents the photonic energy (eV) and ${E}_{U}$ is the Urbach energy quantifying the total energetic disorder of the system. Depending on the metals, ${E}_{U}$ for metals typically ranges between 1 and 10 eV at room temperature^58^. The reciprocal slope of the linear fitting from the plot of ln $\alpha$ (cm^−1^) versus photon energy (eV) gives the ${E}_{U}$ based on the following equation^59^:

10 $${E}_{U}=\frac{1}{slope}.$$

## Results and discussion

### Formation mechanism

The soluble Cu salts inevitably produces two main types of oxide during the Cu oxidation reaction. The two-stage oxide phase formation is based on the sequence of exothermic reactions^60,61^:

11 $$2\mathrm{Cu}+\frac{1}{2}{\mathrm{O}}_{2}\to \mathrm{Cu}_{2}\mathrm{O},$$

12 $${\mathrm{Cu}}_{2}\mathrm{O}+\frac{1}{2}{\mathrm{O}}_{2}\to 2\mathrm{CuO}.$$

The first to the copper oxide or cupric oxide (CuO) nanostructures formation is the deficient cuprous oxide (Cu_2_O). Much information is available that considers the CuO as a product of short oxidation times and the consequences of the steady-state reaction mechanism of the Cu_2_O with O_2_ pressure^62–65^. Principally, the iron-dark colour of CuO in the form of nuclei comes after the formation of the reddish Cu_2_O host and usually settles on the defect surface of the Cu_2_O.

Theoretically, taking the advantages of TA as a modifier and reductant source, the more H^+^ is created through the following reaction process that eases the dissolution of more metal nanostructures^66^:

13 $${M}_{a}{O}_{b}+{\mathrm{H}}^{+}\leftrightarrow {M}^{{(\frac{2b}{a})}^{+}}+{\mathrm{H}}_{2}\mathrm{O},$$

with ${M}_{a}{O}_{b}$ refers to the metal oxide nanostructures and ${M}^{{(\frac{2b}{a})}^{+}}$ is the metal ions freed from nanostructures. The phenolic hydroxyl moieties produced from the deprotonation reaction increases the concentration of H^+^.

**Figure 2:**
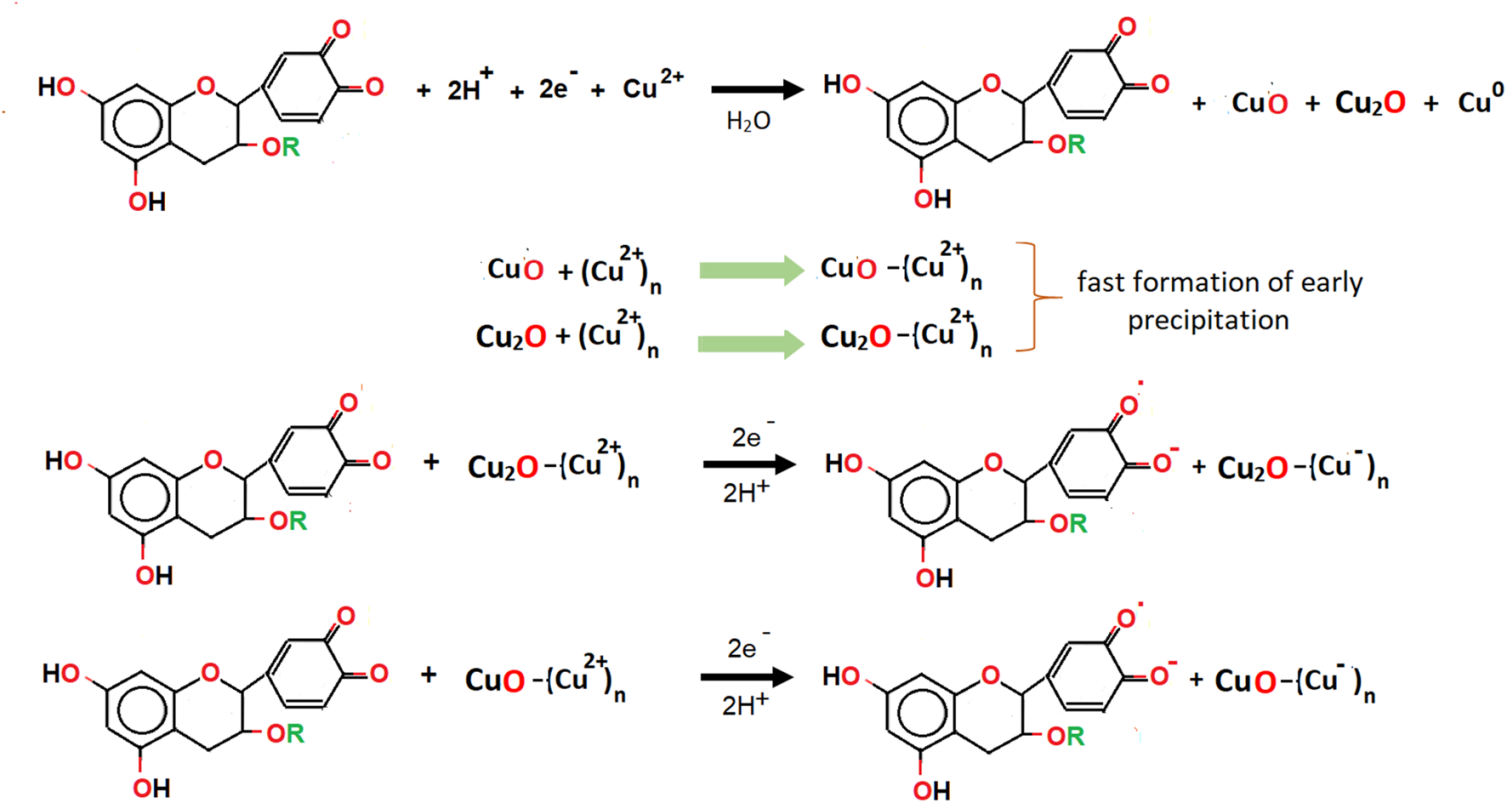
Prediction of pathway mechanisms involved in the formation of Cu-based oxide nanostructures. The reduction of Cu^2+^ is due to the resulting species of –OH link from the quinone in TA.

In the present study, the prediction reaction between quinone of TA and Cu^2+^ is indicated in Fig. 2. The formation of Cu_2_O and CuO nanostructures are the results of electron donation from the quinone segment of TA. In most chemical reactions, it is assumed that only several of the Cu^2+^ are involved in the nucleation of pure Cu as most of them show the existence in the form of Cu_2_O and CuO in the alkaline condition that triggers the formation of these Cu-based oxide nanostructures. At some point, Cu ions integration onto the Cu_2_O or CuO surface is inevitable due to the electron adsorption from the –OH bonding of quinone, causing the production of semi-quinone radical of TA along with electron-rich Cu_2_O- and CuO–(Cu^−^)_n_ species.

**Figure 3:**
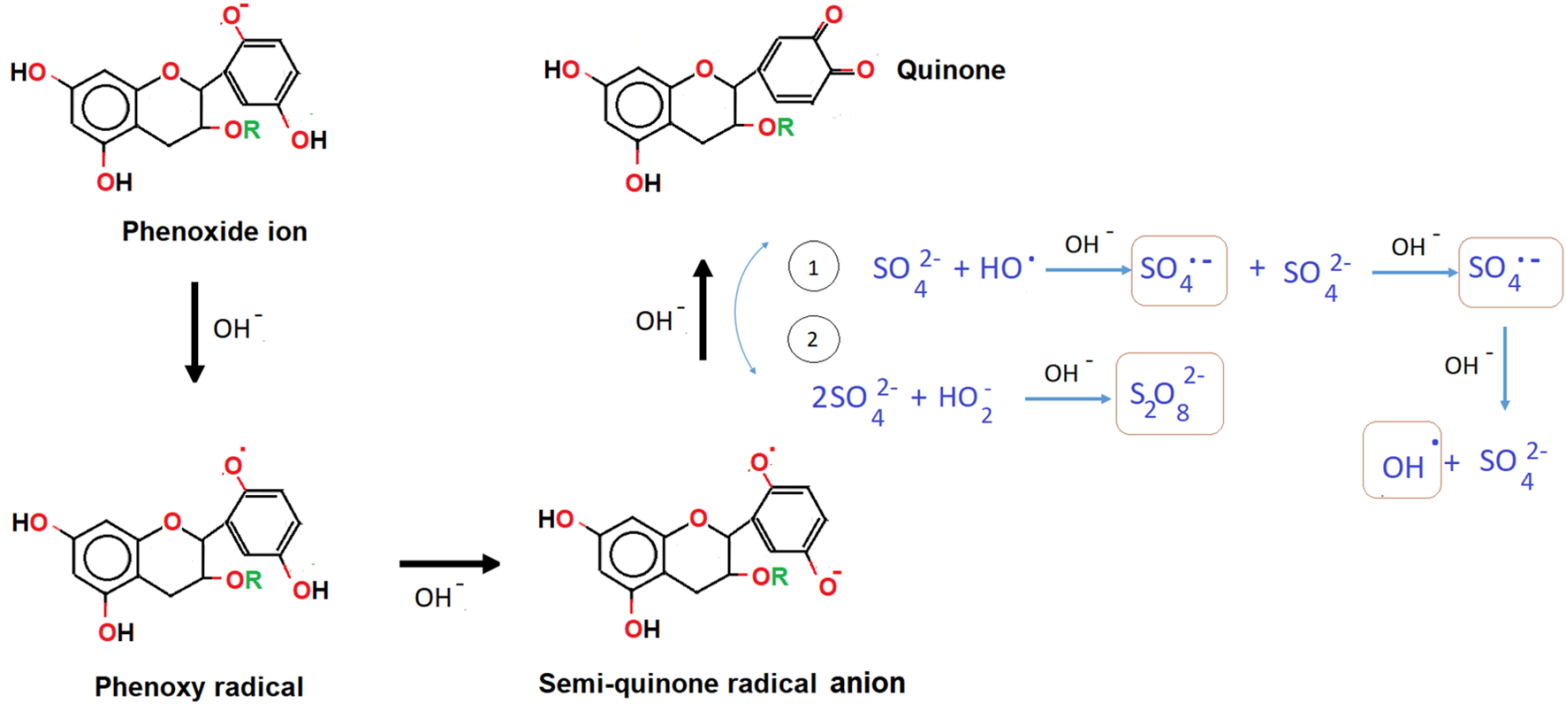
Schematic illustration of the reaction mechanism of phenolic TA from phenoxide ion form to quinone in the presence of sulfate anion from the CuSO_4_ precursor used in the reaction.

As of sulfate anion (SO_4_^2−^), which forms the chain with the hydrated copper ion salt (Cu(H_2_O)_4_) in CuSO_4_ metal salt, the removal of SO_4_^2−^ ion content in the water medium occurs from the anion exchange reaction. The SO_4_^2−^ anions are converted into the persulfate (S_2_O_8_^2−^) anions in the OH^−^ medium as a result of the ionised TA according to the Fig. 3. The ionised TA tends to change to the semi-quinone radical anion which is usually found at pH 7^67^. A characteristic feature of the semi-quinone radicals is that they are not a good oxidant at pH 7. Once the S_2_O_8_^2−^ anions are produced, they react with ionised TA to form the oxidise state of TA, which is the quinone and transform into the sulfate radical (SO_4_^**·**−^). At some point, the SO_4_^**·**−^ then reacts with the remaining SO_4_^2−^ anions to produce more SO_4_^**·**−^. It is noteworthy that high temperature (> 60 °C) also promotes the formation of SO_4_^**·**−^ radicals^68,69^. As more quinone are formed due to the heat activation (in this case is at 80 °C), it causes an increase in the pH (i.e. pH 11) solution. In fact, SO_4_^**·**−^ radicals are easy to convert to hydroxyl (HO^**·**^) radicals under alkaline conditions^70^. Upon completion of the reaction, they are all eliminated from the main solution via centrifugation.

### Morphological and structural properties

**Figure 4:**
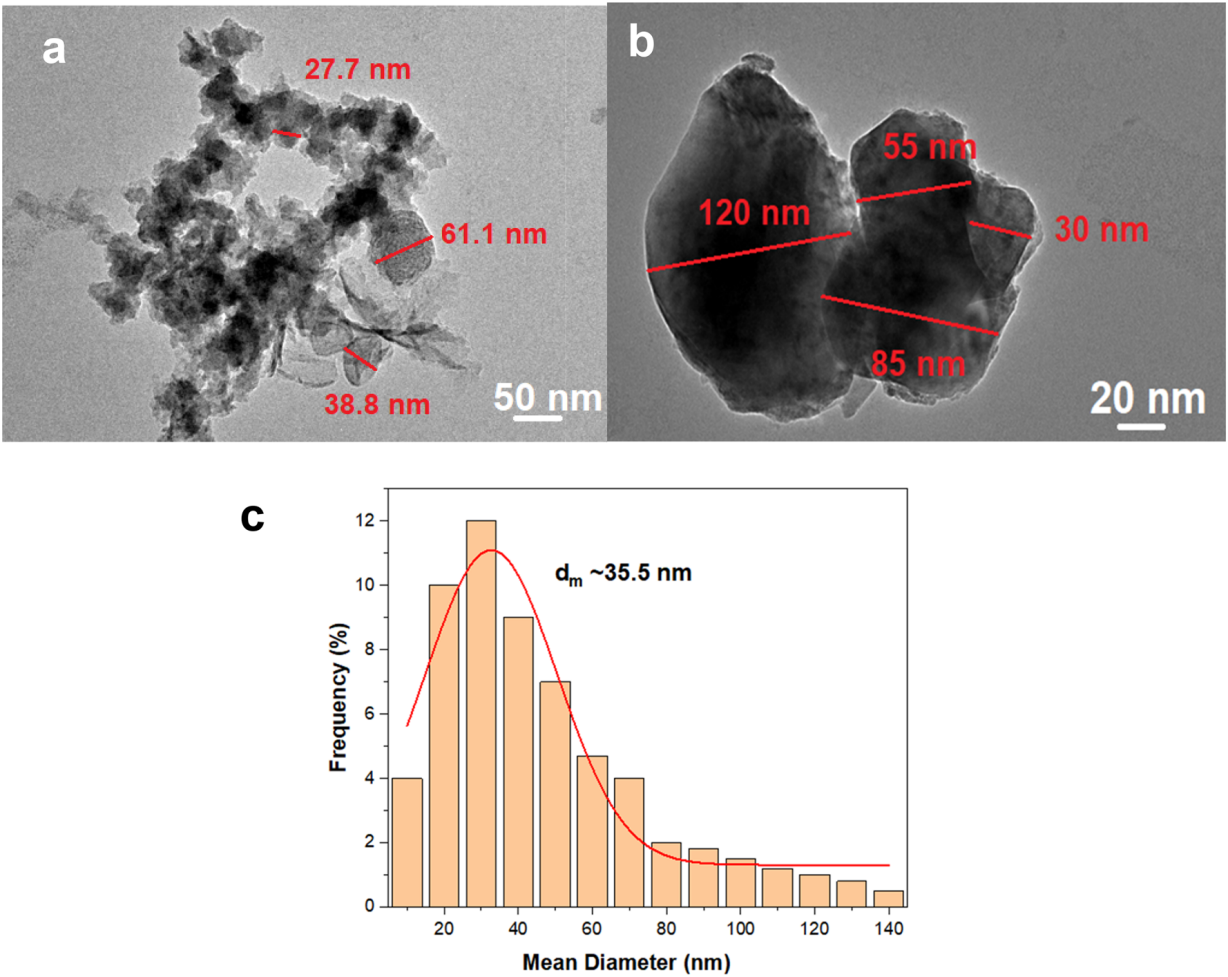
TEM micrographs (**a,b**) and respective histogram (**c**) of the Cu-based oxide nanostructures prepared at 80 °C. The fitting is based on the Gaussian fitting distribution and the ${d}_{m}\sim$ 35.5 nm.

The morphological properties of the as-produced Cu-based oxide nanostructures were resolved by FESEM and TEM analysis. As depicted in Fig. 4, the non-uniform flaky shapes with multiple layers dominate the overall morphology prepared at the highest reaction temperature (80 °C) with pH 11. The average mean particle diameter, ${d}_{m}$ of the flakes for the sample produced at 80 °C is 35.5 $\pm$ 2 nm based on the Gaussian distribution fitting. It is assumed that the variation in the produced shapes, sizes and surface texture depends upon the pH of the TA solution at that particular reactant temperature. The quinones of the TA affect the surface texture of the resulting Cu_2_O and CuO nanostructures, as shown in their aggregated size structure with high probability of interfacial heterojunctions formation. The small flakes, which usually represent CuO morphology, show higher density at the inner area of the center particles with respect to the periphery, proofing unsystematic branched aggregated particle arrangement. Cu atoms released from the step-edge decay process (freed from the oxidation) move to inner surface regions and tend to create aggregation at the thin layer of Cu at the center, rendering the darker region at the designated locality. In this case, the Cu-based oxide nanostructure is mainly dominated by the CuO nanostructure as evident by the existence of more sheet-shape than the pyramidal-shape, which usually belongs to the Cu_2_O nanostructures. The work speculated that the stacking of the flakes is due to the natural chemical adhesions caused by the quinone^71^, which is responsible for the hydrophobic terminated layer surfaces in all the samples. Hydrophobic surface promotes cohesion between the nanostructures^72–74^. This can be visibly proven by the less dispersion and easy sedimentation at the bottom part of the glass vial.

**Figure 5:**
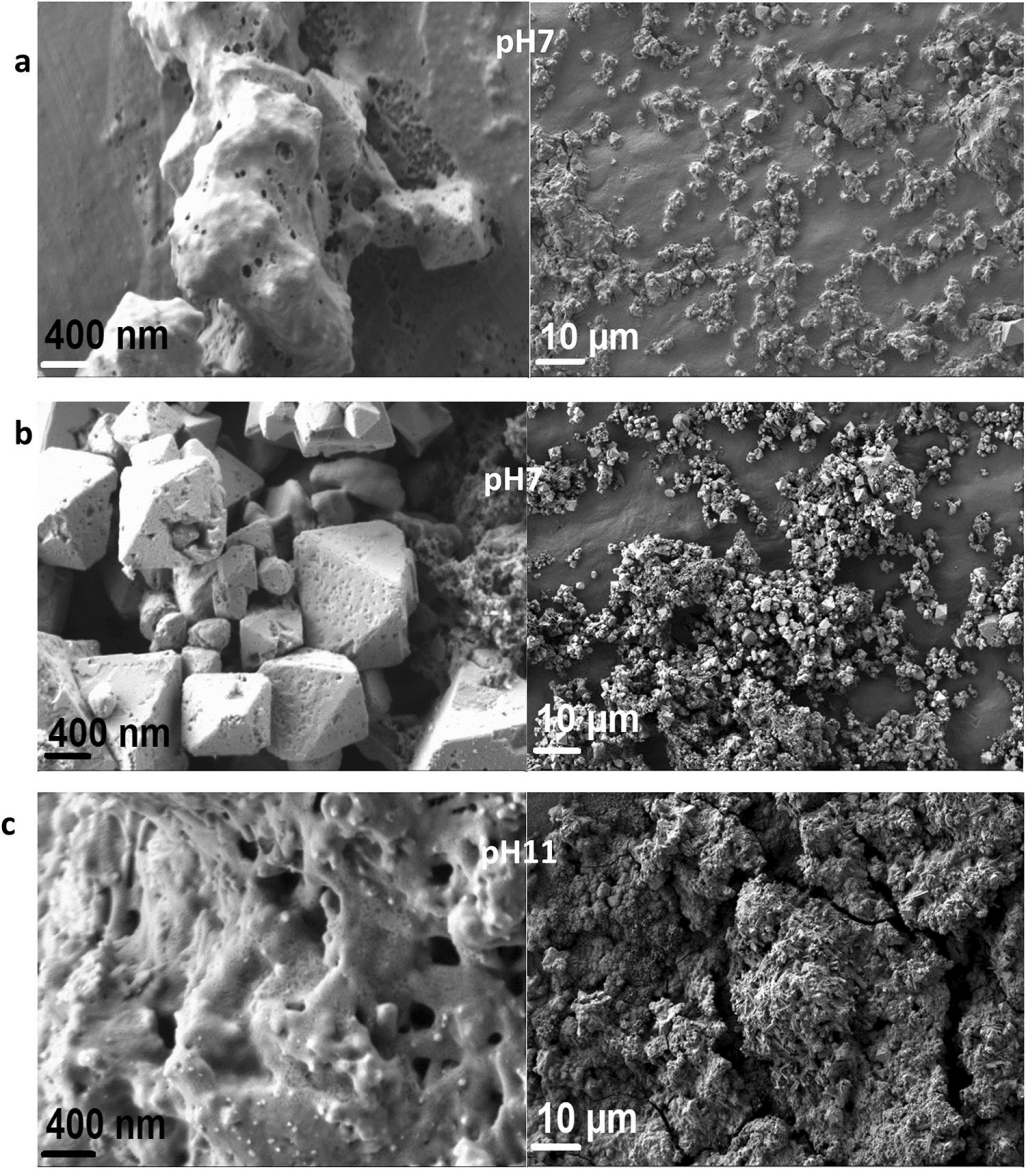
The corresponding FESEM micrographs for Cu-based oxide nanostructures prepared at (**a**) 60 (pH7), (**b**) 70 (pH7) and (**c**) 80 °C (pH11).

The FESEM images of the as-produced Cu-based oxide nanostructures samples are depicted in Fig. 5. As seen in the images, aggregation occurs in all samples. The micrographs show that at 60 °C (Fig. 5a), the sample has protrusions and a rough heterogeneous surface. This indicates that there is a presence of porosity at the lowest reaction temperature with clumpy blended nanostructures assembly. As the temperature increases to 70 °C (Fig. 5b), the aggregated surface of the Cu-based oxide nanostructures becomes smoother and has a denser well-defined pyramidal shape, which reflects more towards the structure of Cu_2_O with a small size protrusion. The dominance of insoluble Cu_2_O nanostructures at 70 °C shows the breakup of TA ligand due to the heat resulting in a decrease of the steric binding site of Cu_2_O-TA. Theoretically, the TA in its weak acidic condition could have five active binding sites per molecule^75^. Hence, the dissociation of the phenolic network promotes the formation of TA radicals leading to a more stable connection with Cu ions. At 80 °C (Fig. 5c), fast coordination rate occurs, yielding rapid Cu-based oxide nanostructures-TA ion mixing and thus causing denser precipitation with high porous surface clumpy-like connected distortion nanostructures. The burning of the branched copper-oxygen polymeric network in high pH conditions results in the formation of porous CuO nanopowder as a solid product. The micrograph shows that the CuO affinity with TA increases based on the presence of the clumps of the aggregation. The temperature is believed to increase the rate of Cu-TA ion exchange which also causes the change of pH values of the system that further influences the CuO and Cu_2_O nanostructures’ precipitation rate. The change in the precipitation rate with temperature is primarily induced by the change in pH and gets accelerated in an alkaline medium.

**Figure 6:**
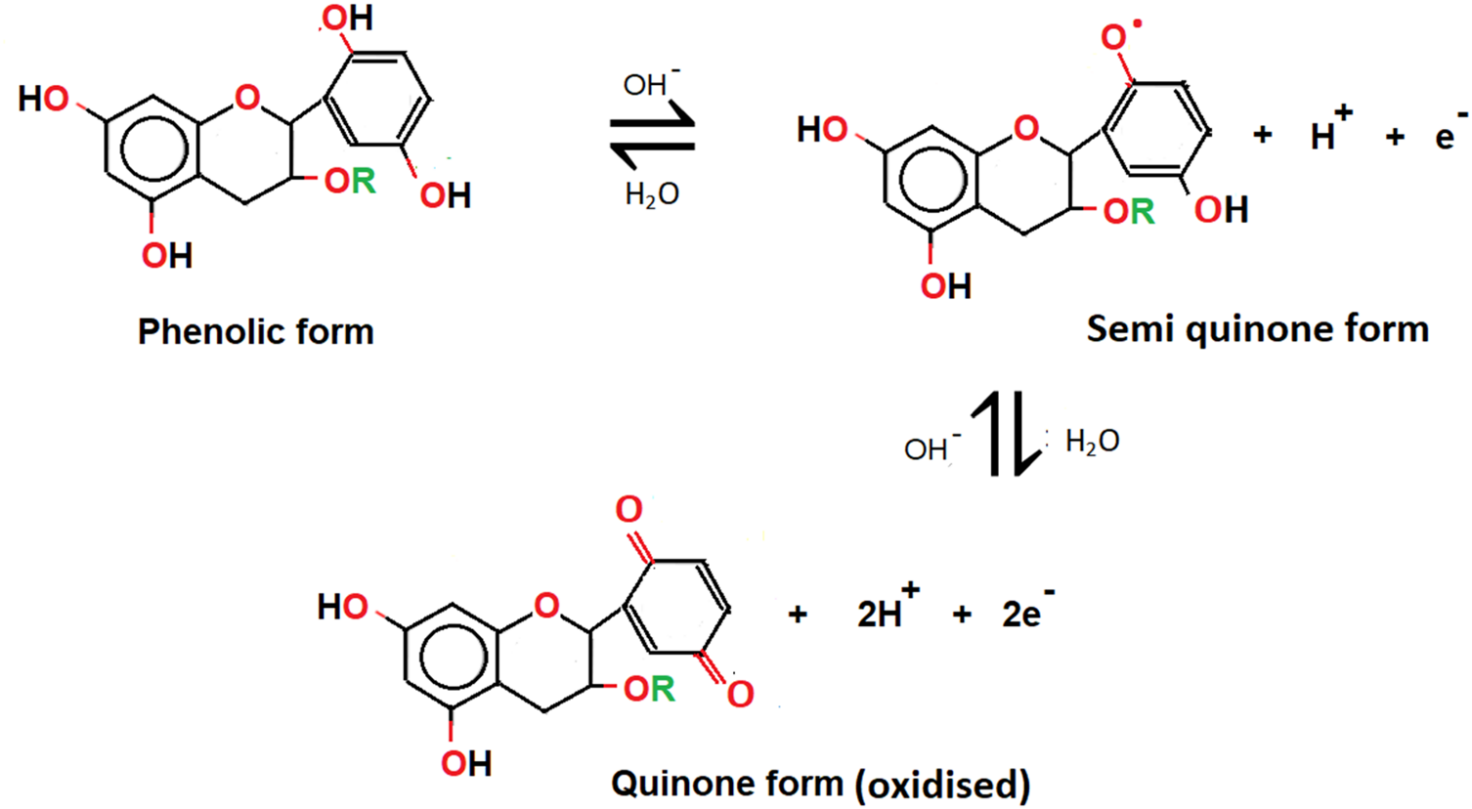
Schematic illustration of the chemical structures of TA’s phenolic, semi-quinone and quinone forms.

As noted, there is a change in the pH value for each produced Cu_2_O and CuO samples. The change in the pH relies on the rate of electrostatic reaction between the TA and the Cu^2+^ ion dissolve that released the Cu-based oxide nanostructures. The weak polyacid (pH < 7) of TA has a negative charge at neutral pH (pH = 7) and thus can interact efficiently with cationic ion Cu for surface functionalization even at room temperature. Nonetheless, the nucleation of Cu nanometal at low pH was not feasible^76^. At low pH conditions, the reaction rates of self-poly-condensations are halted due to the creation of more semi-quinone radicals as the initial oxidation product, whereby the respective rates are sped up at high pH reading. As a result, at high pH conditions, the connected clump with porous Cu-based oxide nanostructures formed that cause by the ‘burning’ of Cu–O from the TA network. Generally, the reduction of Cu ions during the hydrolysis and condensation or oxidation of TA tends to produce semi-quinone and quinone species, respectively (Fig. 6). Under these mild acidic or basic conditions, the TA produces its gallic ester or *d*-glucose structure^77^. The amount of quinone derivative units present as a result of the full oxidation process of TA increases the reduction of Cu ions into Cu metal by donating electrons^78^. Meanwhile, it is known that gallic acid acts as a reducing agent at pH > 7, which guides further formation of Cu-based oxide nanostructures and Cu metal. Even so, gallic acid is a weak stabilizer at this pH and caused the Cu-based oxide nanostructures to suffer from aggregation. In contrast, glucose role at alkaline pH is a replication of stabilizing agent but a poor reducing agent^76,77,79^. It can be seen that the connected clumped nanostructures with the unstructured morphology are much larger at 80 °C as compared to 60 and 70 °C. These contribute to the changes of pH from 7 to 11 as high temperature will cause more production of reactive quinones of TA and thus generate more amount of gallic acid in response to the increase of oxygen release (oxidation process)^80^. The quinone species are reported to possess strong alkaline pH^81^. The inevitable increase in the alkalinity is led by the rise in the galloyl semi-quinone radicals from the TA hydrolysis reaction (molecule coordination). The condition further gives rise to a self-crosslinking reaction between the Cu ions and TA and contributes to the formation of the bis-complex CuO or Cu_2_O aggregates. The increase in the TA composition on the surface of CuO or Cu_2_O promotes the increase in the amount or size of the more prevalent protrusion via protonation reaction and deteriorate the surface structures.

**Figure 7:**
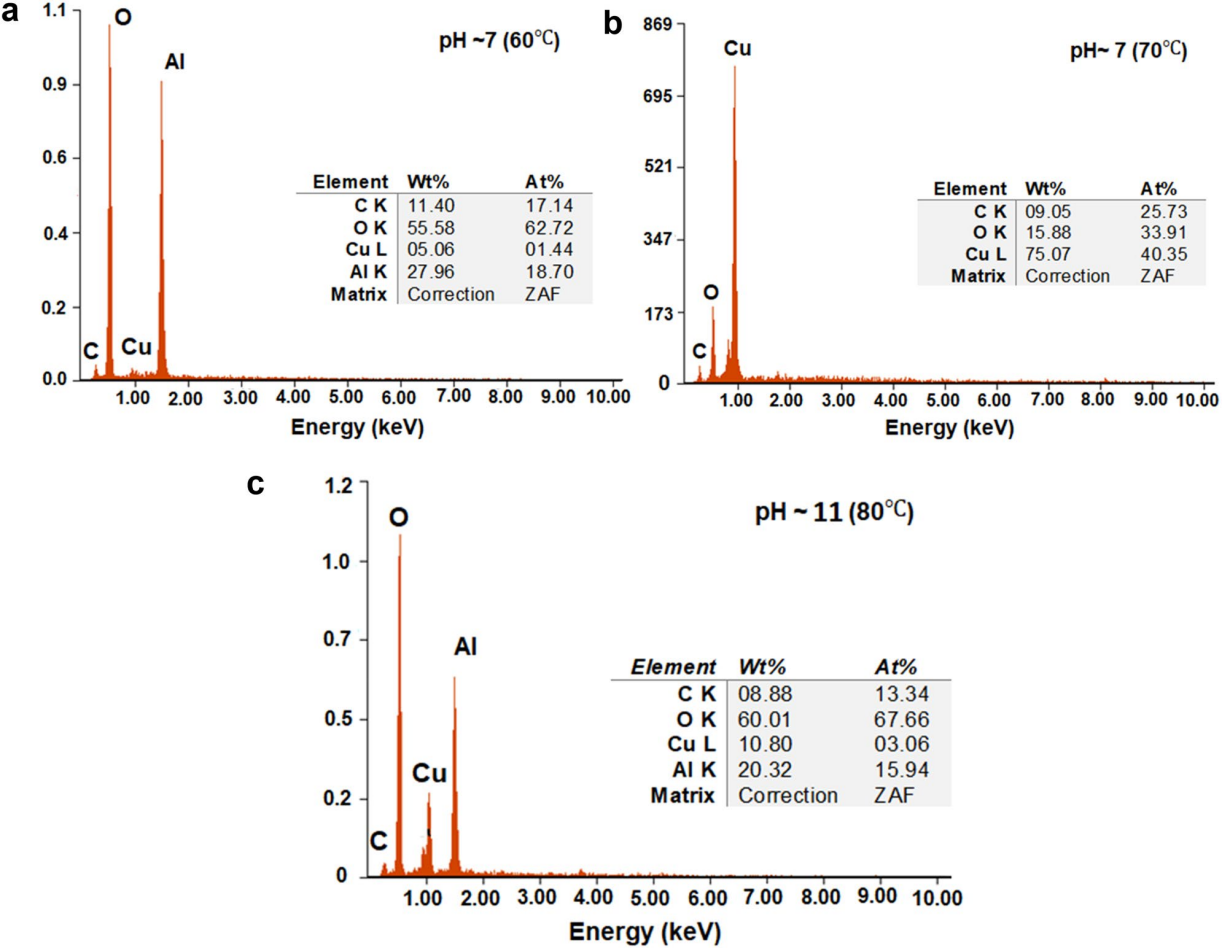
The corresponding EDX analyses for Cu-based oxide nanostructures prepared at (**a**) 60, (**b**) 70 and (**c**) 80 °C.

The corresponding EDX in the selected sites for the as-synthesised samples at 60, 70 and 80 °C are shown in Fig. 7. The mapping images show a significant difference in the distribution of the chemical elements for each sample. All the samples show the common elements, C and O, which can be found in the TA structure apart from the Cu element. It is assumed that the TA is adsorbed on the surface of Cu-based oxide nanostructures with incomplete surface coverage. Also, there is an exceptional signal corresponding to present of Al due to the usage of aluminium foil as the substrate for the FESEM analysis. Those associated Al signals are strong in the 60 °C (Fig. 7a) and 80 °C (Fig. 7c), which confirms the dissociation of CuO (weak signal of the sample as compared to the substrate) as noted in the atomic percentage of elements contained in the functionalised Cu-based oxide nanostructure samples. Both samples indicate severe oxidation of the CuO nanostructures. Only sample at 70 °C indicates high purity of Cu element (Fig. 7b). This proves the discussion on the high crystallinity of the well-defined pyramidal shape of Cu_2_O nanostructures morphologies from Fig. 5b discussed previously. Although TA possesses a high phenolic C–C bond, the C content is low, indicating the sample has a stable radical species and clean from impurities. The high percentage of O is due to the increase in the interaction (cation bridging) between phenolic O–H moieties and the Cu nanostructure surface.

Furthermore, as per the EDX analysis results, the as-produced Cu-based oxide nanostructures are shown to have almost pure CuO at pH7 (60 °C), pH 11 (80 °C) and Cu_2_O is the primary substances at pH 7 (70 °C) as indicated in Table 2. The atomic percentage of oxygen is far higher at pH7 (60 °C $$)$$ and pH 11 (80 °C) due to the high amount of quinone presence that led to the high oxidation rate. These high differences contributed from the formation of stronger and prominent bonding between Cu and higher added oxygen elements (adsorption) in the samples, especially at pH11. Beyond pH7, the TA is ionised and fully oxidised, resulting in the formation of strong Cu-based oxide-TA nanostructures. Hence, an alkaline pH medium stimulates the generation of coagulated and homogenised suspension. Yet, for pH 7 (70 °C) the percentage of Cu is slightly greater than that of O as a result of complete diffusion and adequate balance bonding between the Cu and O. Overall, the decrease or increase of the oxygen percentage is due to the conversion rate of the phenolic to quinone of TA as shown in the reaction flow beforehand. Meanwhile, the percentage of Cu represents the amount of Cu nucleation via the conversion of Cu^2+^ to Cu^0^ using the denoted electrons from the quinone of TA. At this stage, the comparison between the pH 7 at 60 and 70 °C can be done based on the role of reaction temperature which affects the secondary binding of the quinone^82^. It shall be noted that the relative ratio calculated from the atomic percent values is in accordance with the expected theoretical percentages of Cu and O elements in CuO and Cu_2_O nanostructures.

**Table 2:** The Cu to O ratio is based on the atomic percentage obtained from the EDX results.

Sample	Primary substance	Atomic % from EDX		Cu:O
		Cu%	O%	
pH 7 (60 $^\circ{\rm C}$)	CuO	1.44	62.72	1:43
pH 7 (70 $^\circ{\rm C}$)	Cu_2_O	40.35	33.91	1.12:1
pH 11 (80 $^\circ{\rm C}$)	CuO	3.06	67.66	1:22

**Figure 8:**
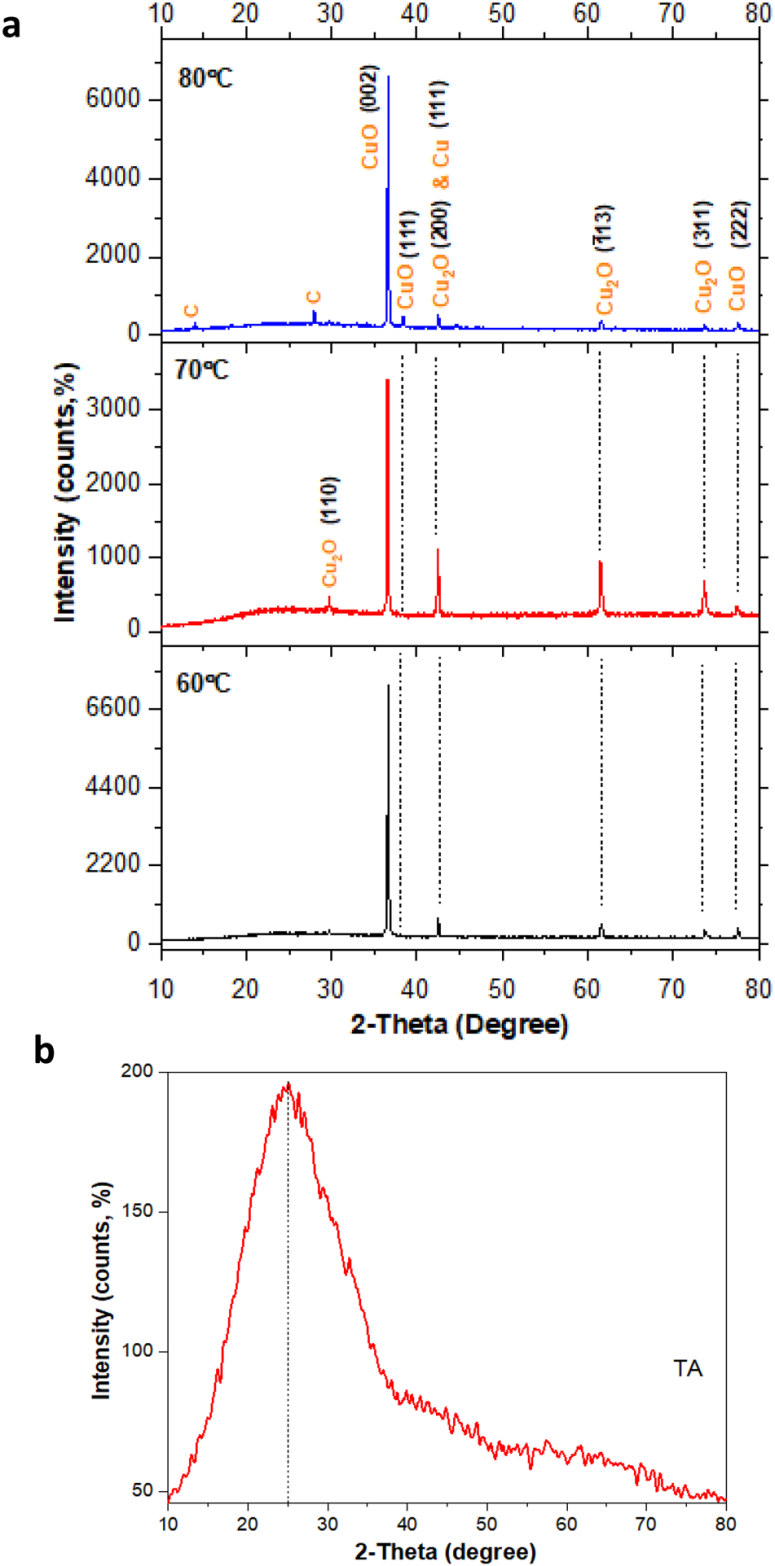
The XRD pattern of Cu-based oxide nanostructures prepared at (**a**) 60, 70 and 80 °C with the (**b**) TA pattern for comparison.

The crystallinity phase of the samples is determined via the XRD analysis. It is interesting to note that different crystalline phases are present in each sample as shown in Fig. 8a. Although at earlier assumptions, the work suggests that only CuO and Cu_2_O are present, the pattern also shows the existence of a small amount of crystalline Cu nanostructures in all samples. It is fascinating to assume that the synthesis produced a mixture of pure metallic Cu and its Cu^+^ states of nanostructures (monoclinic CuO and cubic Cu_2_O). The sample at 60 °C shows the sharpest and narrower peaks of the CuO phase. The oxidation of Cu-based oxide samples is evident by the presence of the most significant lattice planes of CuO at 2 $\theta =$ 36.6° assigned to (002) in all samples, which also serve as preferential oxygen absorption sites among other peaks. The preferential planes at (111) and (222) are also found for CuO substance in all samples, corresponding to the resulting oxidation in single oriented planes. The corresponding XRD peaks assigned to (111) and (200) with a low peak intensity for pristine face-centered cubic pure Cu and Cu_2_O can only be seen at 2 $\theta =$ 42.4° for all sample. Notably, the oxidation process proceeds which causes a faster oxidation reaction with a weak diffraction peak of pure Cu appearing and overlapping the peak of Cu_2_O phase at similar 2 $\theta$ reading and at the same time produced stronger diffraction peak intensity of CuO phase. Meanwhile, minor peaks of Cu_2_O also found in all samples assigned to the (200), ($\overline{1 }13$) and (311) at 2 $\theta =$ 42.4, 61.6 and 73.7°, respectively. For sample at 70 °C, it is believed that the oxidation process is lesser than the 60 $^\circ{\rm C} ,$ which results in the increase of the intensity diffraction peak of the Cu_2_O phase. The minor trace of the C phase at 80 °C is believed to be strongly contributed by the TA in the system (2 $\theta =$ 14.8°). As indicated in Fig. 8b, the actual TA data presented the broad peak at the value of 2 $\theta =$ 25° also proves that the occurrence of the amorphous phase of TA does not show any discernible reflection pattern. Therefore, it is evident that the temperature could easily alter the pH values and thus affect the Cu system’s oxidation level.

In addition, the lattice parameters of Cu_2_O and CuO nanostructures that demonstrated cubic and monoclinic crystal lattices, respectively, are calculated based on the lattice geometry equations and are listed in Table 3. The unit cell volume, $V$ variation with respect to pH also indicated various types of Williamson–Hall strain values in the crystal structure, which is discussed later. As demonstrated in Table 3, the lattice parameters and volume unit cell in CuO at pH7 (70 °C) is the highest representing the grain development compared to the other two samples. As for Cu_2_O, the highest lattice parameter of *a* is observed at the ($\overline{1 }$13) plane in all samples indicating the primary lattice plane occupied by the Cu_2_O in each sample. The reported lattice parameter data are consistent within the range but slightly different from the literature data collected for CuO^39,83,84^ and Cu_2_O^85,86^. These are attributed to the influence of the types of synthesis route used and the chemical reagents employed in the reactions, which further indicate different constraints. Each synthesis processes produced varied results and to gain the exact and uniform morphology with detailed lattice indexes is trivial due to the various steps involved, which might complicate the control of the morphology and uniformity of the particle sizes.

**Table 3:** Crystallographic parameters consist of the lattice parameters and unit cell volume of CuO/Cu_2_O nanostructures with the wavelength of Cu Kα radiations at $\lambda =$ 1.5406 Å.

Sample	Substance	*2* $\theta$(°)	$\theta$(°)	Miller indices			*d*_*hkl*_ (Å)	Lattice parameters (Å)			*V*(Å^3^)
				*h*	*k*	*l*		*a*	*b*	*c*	
pH7 (60 °C)	CuO	36.63	18.31	0	0	2	2.4508	–	–	4.9016	94.7002
		38.05	19.02	1	1	1	2.3630	4.5507	–	–	
		77.58	38.79	2	2	2	1.2294	–	4.2986	–	
	Cu_2_O	42.52	21.26	2	0	0	2.1242	4.2485	–	–	76.6872
		61.59	30.79	$\overline{1 }$	1	3	1.5043	4.9895	–	–	124.214
		73.64	36.82	3	1	1	1.2852	4.2625	–	–	77.4488
pH7 (70 °C)	CuO	36.57	18.28	0	0	2	2.4550	–	–	4.9101	100.386
		38.28	19.14	1	1	1	2.3493	4.7568	–	–	
		77.54	38.77	2	2	2	1.2300	–	4.3532	–	
	Cu_2_O	28.26	14.13	1	1	0	3.1545	4.4612	–	–	77.3397
		42.46	21.23	2	0	0	2.1269	4.2538	–	–	124.547
		61.53	30.76	$\overline{1 }$	1	3	1.5057	4.9939	–	–	76.9769
		73.68	36.84	3	1	1	1.2846	4.2605	–	–	88.7881
pH11 (80 °C)	CuO	36.63	18.31	0	0	2	2.4510	–	–	4.9020	94.9374
		38.40	19.20	1	1	1	2.3422	4.5538	–	–	
		77.59	38.79	2	2	2	1.2294	–	4.3062	–	
	Cu_2_O	42.51	21.25	2	0	0	2.1245	4.2491	–	–	76.7205
		61.59	30.76	$\overline{1 }$	1	3	1.5057	4.9939	–	–	124.232
		74.54	37.27	3	1	1	1.2719	4.2187	–	–	75.0841

**Figure 9:**
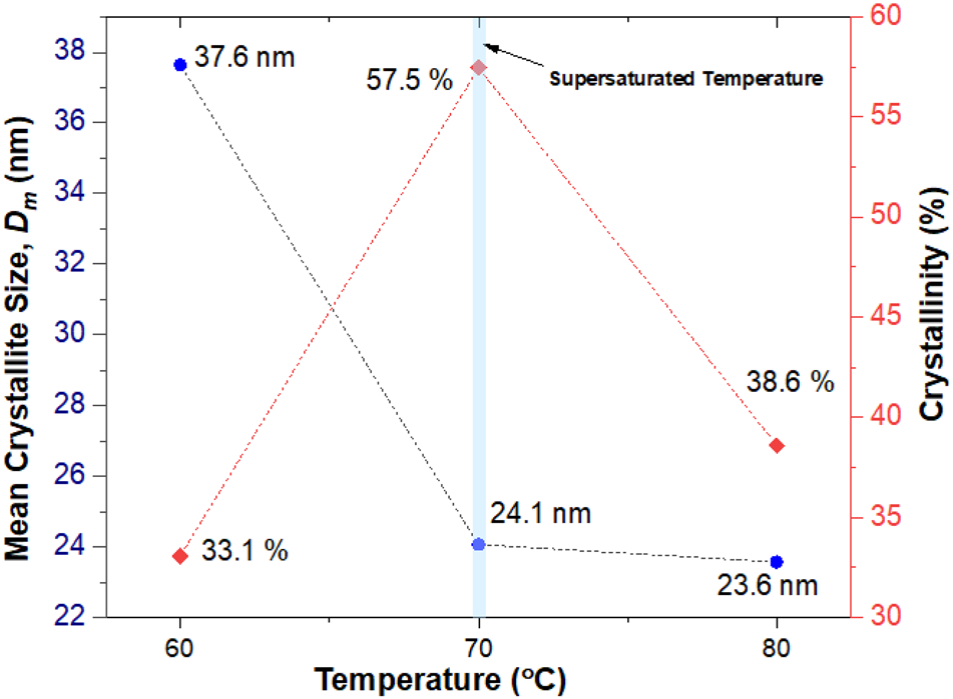
The mean crystallite size, ${D}_{m}$ and percentage crystallinity calculated from XRD data.

Based on Fig. 9, the ${D}_{m}$ patterns for these three samples are quite linear, where there is a gradual decrease in the ${D}_{m}$ as the reaction temperature increased from 60 to 80 °C. The decrease in the ${D}_{m}$ is caused by the formation of the extra element of C due to the strong influence of TA in the sample at 80 °C (Fig. 8a). The difference in the ${D}_{m}$ between sample at 70 °C ($\sim$ 24.1 nm) and 80 °C ($\sim$ 23.6 nm) are not significant as compared to the 60 °C ($\sim$ 37.6 nm) sample. It is believed that high reactant temperature coupling with natural enzymes of TA favours much higher oxidation through the oxygenation reaction. This could be attributed to the enhanced mobility or diffusion rate of the Cu oxyanions at 70 °C and 80 °C, which consequently increase the contact between the Cu ions and the adsorption sites of TA in the system. Although the activation energy for bulk CuO and Cu_2_O formations is above 300 °C^87,88^, in the presence of TA, the formation of these nano-oxides is relatively faster which explains the onset of reaction to occur below 100 °C. The aggregates’ formation that affects the cluster formation can also be affiliated to the increase in the surface roughness triggered by the pH migration to the alkaline region of the Cu-based oxide–TA nanonetwork structures.

As previously noted, the reaction temperature influences the pH and thus controls the size/morphology of the nanostructures as evinced by the FESEM micrographs. With the changes in the temperatures, the establishment of new morphologies and different dispersibility states manifest. It is worth mentioning that the temperatures alter the pH and affect the crystalline quality of the as-produced nanostructures. The crystallinity is a factor that can only be gained from the FWHM data and reflects the strain contribution which controls the broadening of the peak. It is seen from the FESEM micrograph of 70 °C that a well-defined nanostructures were present, and this is evident by the 57.5% of crystallinity indicated in Fig. 9. By comparison, the percentage crystallinity of the samples at 60 and 80 °C are 33.1 and 38.6%, respectively. The lowest crystallinity is at 60 °C, despite the ${D}_{m}$ at this temperature is the highest among them all. In regards to this, there is no ideal relationship scheme of changes between the temperature and crystallinity or ${D}_{m}$ for the system. One can consider that the Cu species creates the particles in conjunction with their chemical behavior (i.e.*,* due to the variation in the pH) of the size groups (i.e., for colloids or particles is considered large size). In addition, at 60 °C with pH7, the polyhydric phenol is oxidised to quinone and the quinone leads to the opening of pores on the surface, which resulted in the increase in specific surface area by creating a more interconnected structure. The Cu ions become more concentrated and create a large crystallite size. For the sample prepared at 70 °C, the sample remains at similar pH. However, the activation condition continues. The diffusable phenolic and quinone contents facilitate the oxygen-rich condition (denotes electrons), thus yielding a smoother surface structure with higher crystallinity percentages. The crystallinity or crystal number increases linearly with the supersaturation effect and 70 °C at pH7 is assumed to be the supersaturated point, which estimates the metastable region. The number of crystals produced is a notable effect by the pH but not on the temperature. The pH increased to 11 at 80 °C, a significant drop to 38.6% of crystallinity with no exact shapes of structures is observed. The effect of cross-linked quinone on the crystallinity of the CuO at 80 °C in comparison to 60 °C indicates the role of pH in influencing the degree of crystallinity. A slightly high percentage of crystallinity at pH11 as compared to pH7 is due to the presence of more cross-linked quinone. As noted earlier, the transition to alkaline pH causes high production of reactive quinones of TA and thus generate more amount of gallic acid in response to the increase of oxygen release (oxidation process). At this point, the Cu-based oxide-TA nanonetwork complexion at alkaline pH constitutes lesser porosity with considerable increase of heterogeneous coagulation clump (increasing density). Although these samples are interpreted as polycrystalline samples based on the ordered nanostructures 80 °C TEM images, samples at 60 and 80 °C are proved to have “low crystalline quality”. Hence, the strongest contributing factor influencing the crystallisation percentage is the pH, controlled by the temperature in the present case.

**Figure 10:**
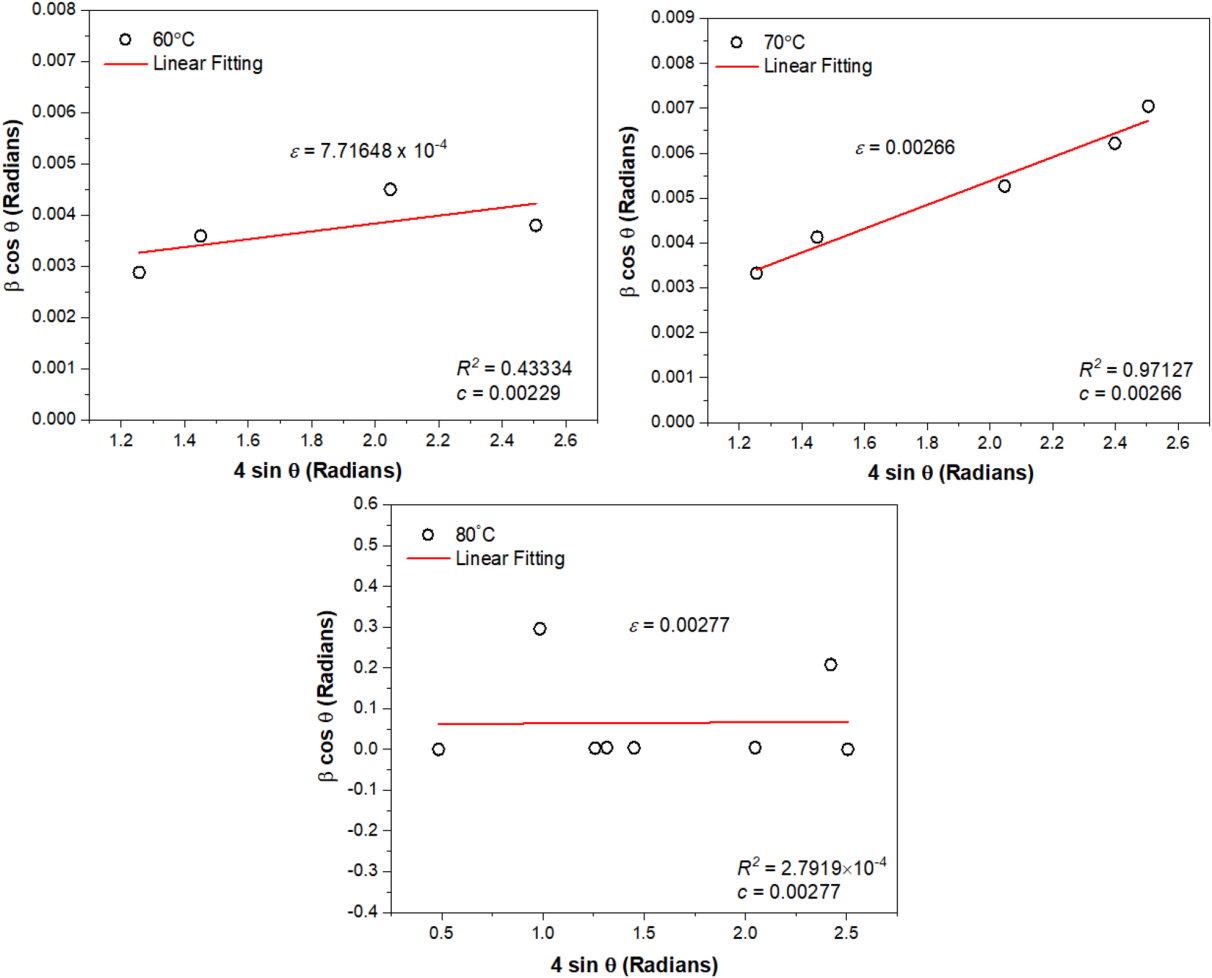
Linear fitting calculated using W–H correlation for Cu-based oxide nanostructures prepared 60, 70 and 80 °C. The slope indicates the mean microstrain, $\varepsilon$ and the intercept line at the y-axis, *c* manifest the inverse of ${D}_{m}$.

For crystallinity percentage, it is believed that the condition is created by elastic and inelastic scattering of the sample. Both the scatterings contributions are able to give extra information on the nanostructural parameters. Williamson–Hall (W–H) plot is able to bring out the individual characteristics of nanostructure size and the lattice microstrain that exist based on the broadening of the peak in the samples. The plot uses the FWHM adjustment or integral breadth, $\beta$ values, which set apart the size and microstrain effects according to the separation of their unique diffraction angle, *θ*. These results act as the additive on the $\beta$ and their FWHM characteristics. W–H plots for 60, 70 and 80 °C are inscribed in Fig. 10. These samples are considered to consist of microstrain-free crystals with minimum lattice strain (trend line slope) values below 0.003%. These very low lattice microstrain might cause by the liquefying process on the formation epitaxial fusion surface of nanoparticles subunit. The occlusion causes the defect or deformation in the lattice structure. The observation on the percentage of the microstrain shows an increasing trend from $\varepsilon =$ 7.72 $\times 1{0}^{-4}$, 0.00266 to 0.00277 as the crystallite size decreases from 37.6 nm (60 °C), 24.1 nm (70 °C) and 23.6 nm (80 °C), respectively. The positive values of the $\varepsilon$ prove the tensile strain condition dominates the sample and not the compressive strain, which usually shows the negative values. The linear regression fit was found to be the best-fit line for 70 °C (R^2^ = 0.97127), followed by 60 °C (R^2^ = 0.43334). The crystallite size is the smallest when the slope is approximately zero as inscribed by 80 °C (R^2^ = 2.7919 $\times {10}^{-4}$) where the fit is $\sim$ 0. Meanwhile, the intercept, *c* is inversely proportional to the size of the domain. These data demonstrated that the primary source of line broadening is the microstrain that originates from the occlusion of organic matter, the TA within the nanocrystal structures and not domination from crystallite size, ${D}_{m}$.

### Optical properties

**Figure 11:**
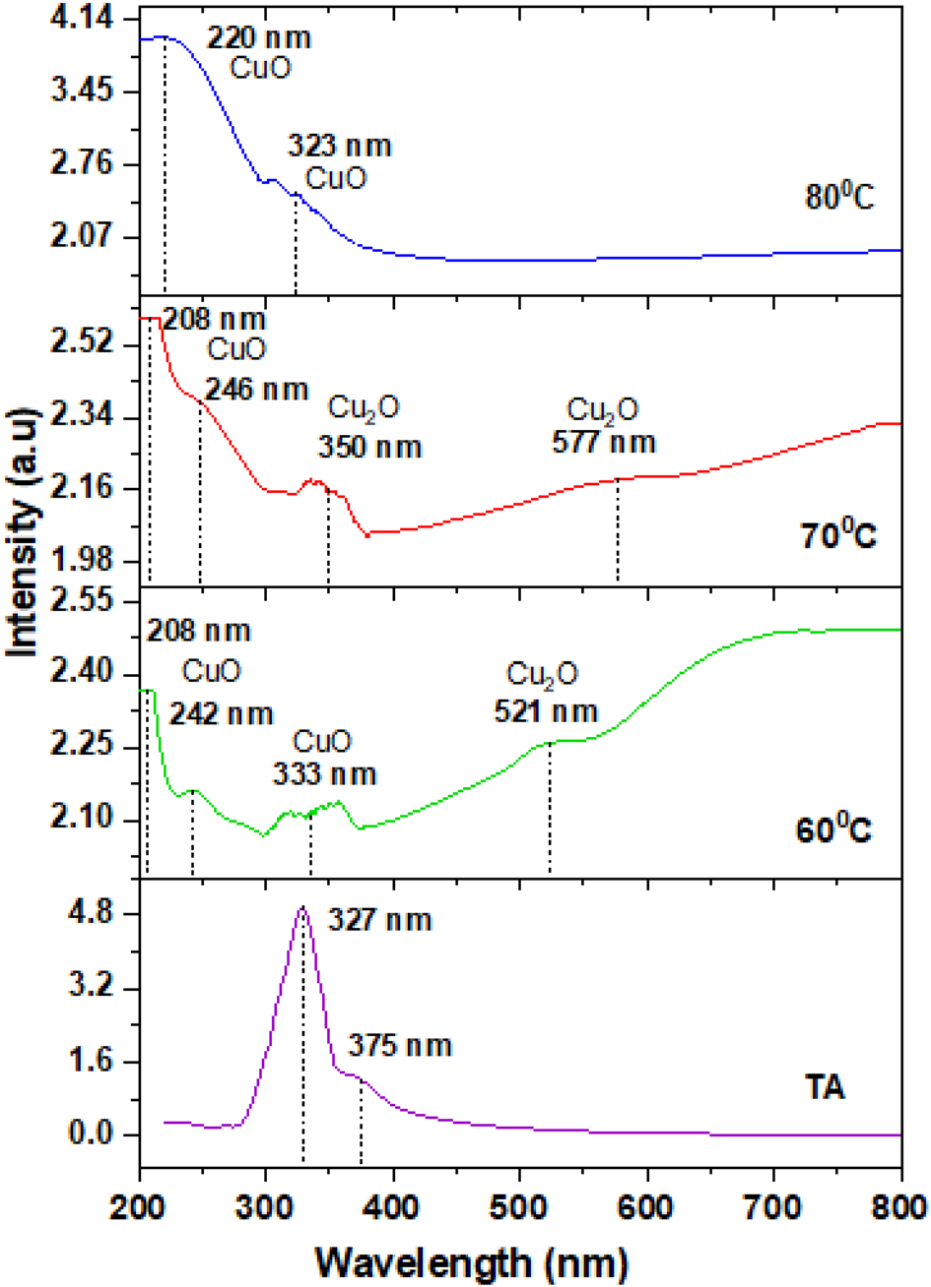
Absorbance spectra of the TA, the as-produced of CuO and Cu_2_O nanostructures prepared at 60, 70 and 80 °C.

Figure 11 indicates the UV–Vis absorbance spectra of the Cu-based oxide nanostructures and the spectrum of TA. The TA has the highest absorbance peak at 327 nm. The absorption peaks of Cu-based oxide nanostructures are found in the range of 220 to 233 nm and 350 to 577 nm for Cu_2_O and CuO nanostructure peaks, respectively. The CuO shows the absorbance range at 220 to 246 nm in all samples^89^. At 60 and 70 °C, the Cu_2_O nanostructures exhibit absorbance peaks located at the range of 350 to 577 nm^90,91^. The highest intensity peak for the sample at 60, 70 and 80 °C appears at 208, 208 and 220 nm, respectively. At 80 °C the peak at the lowest and highest wavelengths diminish and the remaining two peaks of CuO nanostructures are blue-shifted as compared to the sample at 60 °C, which signifies the decrease in the average particle size. The significant variability of UV–Vis peak between 60 and 80 °C is shown by the diminishing of 208 nm CuO peak that presents at 60 °C but disappears at 80 °C, apart from the absence of Cu_2_O peak at 521 nm. These two CuO peaks from sample 60 °C located at 242 and 333 nm are shifted towards the lower wavelength (i.e*.* 220 and 323 nm), respectively, for 80 °C. Conversely, a red-shifted absorption peak was observed for samples at 60 to 70 °C (i.e. CuO from 242 to 246 nm; Cu_2_O from 521 to 577 nm). This confirms the crystallite size pattern obtained from the Scherrer equation beforehand. It is strongly believed that there is no significant trace of pure Cu metal peak in the spectra as the Cu nanostructures possess a clean and sharp peak of absorbance peak at the range of 300 to 600 nm^92,93^.

**Figure 12:**
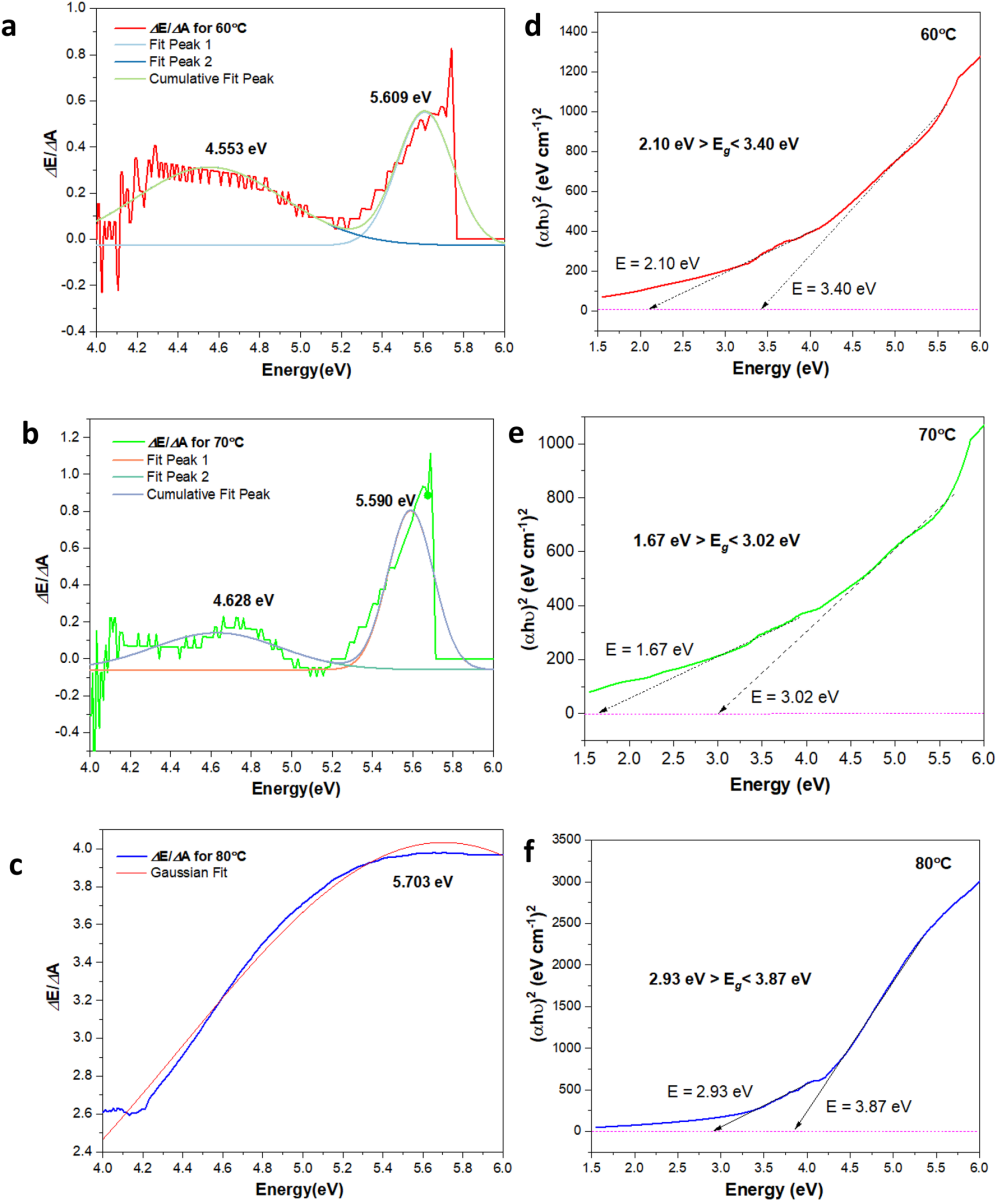
Optical bandgap determination from energy derivative of the absorbance curve, $\frac{\Delta E}{\Delta A}$ (left) and Tauc bandgap for indirect photon energy (right) using the estimate of direct energy bandgap ($\alpha h\upsilon {)}^{2}$ *vs* energy (eV) plot for (**a,d**) 60, (**b,e**) 70 and (**c,f**) 80 °C.

The variation in the absorption intensity is due to the synergistic effects of the low absorption of Cu-based oxide nanostructures and the high absorption of TA. The synergistic effect can be showcased by increased microstrain as the absorbance profile shifts to the blue region. The results are in accordance with the W–H strain plot discussed previously. It shall be noted that there exists a relation between the increase of the microstrain of the system with the bandgap energy, which eventually correlated to the ${D}_{m}$. The difficulty arises as the samples consist of both the Cu_2_O and CuO nanostructures. Since the Cu-based oxide nanostructures consist of both the ${E}_{g}$ for Cu_2_O and CuO, it is pretty worth finding out their corresponding multi-band gap of the samples. The maximum in the derivative spectrum at the lower sides of the ${E}_{g}$ (longer wavelength) represent the multi-band gap with the direct and indirect transition of the samples. Here, the derivative absorbance with reference to the photon energy (*E*) is a qualitative analysis and quantification method that is more complex than zero-order spectra. The first-order derivative is used to attain the width of the forbidden band, ${E}_{g}$ of this Cu-based oxide nanostructures from the absorption data. As can be seen from Fig. 12a–c, the lowest range has many errors mainly due to data dispersion and the reliable ${E}_{g}$ range only starts at 4.2 eV instead of zero. The multi-band gaps for 60, 70 and 80 °C show the lowest side of ${E}_{g}$ at 4.55, 4.63 and 5.70 eV, respectively. The values suggest the corresponding derivative absorbance owing to the varied characteristics in the nature of the multi-${E}_{g}$. Nonetheless, the high effect of TA that they experienced makes the samples exhibit different crystalline structural quality of Cu-based oxide nanostructures.

Meanwhile, the direct Tauc ${E}_{g}$ is focused only on the CuO nanostructures (the highest compound element). The present work also considers the calculation of the indirect Tauc bandgap, but the values obtained fall in the range of negative ${E}_{g}$ values, which is not reliable and the work omits the results. Figure 12d–f shows the Tauc plots of the samples prepared at 60, 70 and 80 °C. The extending linear segments of direct energy bandgap (*αhυ*)^2^ versus the photon energy (eV) plots are calculated. Based on absorbance data for 60, 70 and 80 °C$,$ the Tauc ${E}_{g}$ are in the range of 2.10 to 3.40 $\pm\, 0.1$, 1.67 to 3.02 $\pm \,0.1$ and 2.93 to 3.87 eV$\pm\, 0.1$, respectively. The bandgap trend shows that the range values increase with increasing temperatures. As the values are correlated with the ${D}_{m}$, it inscribed that the ${E}_{g}$ values increase with decreasing ${D}_{m}$.

Also, the pH alters the absorbance spectra and the corresponding changes in ${E}_{g}$ are notable. The pH mainly influences the natural condition of these Cu-based oxide nanostructures and directly affects spectral changes according to the basic or acidic surface environment. As noted beforehand, the values gained for multi-${E}_{g}$ and the Tauc ${E}_{g}$ indicate dissimilarity with the multi $-{E}_{g}$ presenting considerable error values. It seems that the direct Tauc method might be least accurate in deciding the multi-${E}_{g}$ and frequently yield inaccurate conclusions in examining the changes of the ${E}_{g}$. In most cases, the Tauc method is suitable to estimate the ${E}_{g}$ of the bare and single element material. Unlike the first-order derivative approach, the inherent disorder (cause the microstrain) that causes the deconvoluted spectrum contributes to the data dispersion, further indirectly gives more feasible data. To get a more accurate estimation of the results, the use of baseline fitting in the first-order derivative approach provides the complex excitation energy of the system. The baseline fitting approach is more reliable and gives satisfying results. Nonetheless, the measurement’s sensitivity is also increased, which could be contributed by the differences in measurement-analyses condition (e.g., fitting range, optical misalignment and spectra correction).

**Figure 13:**
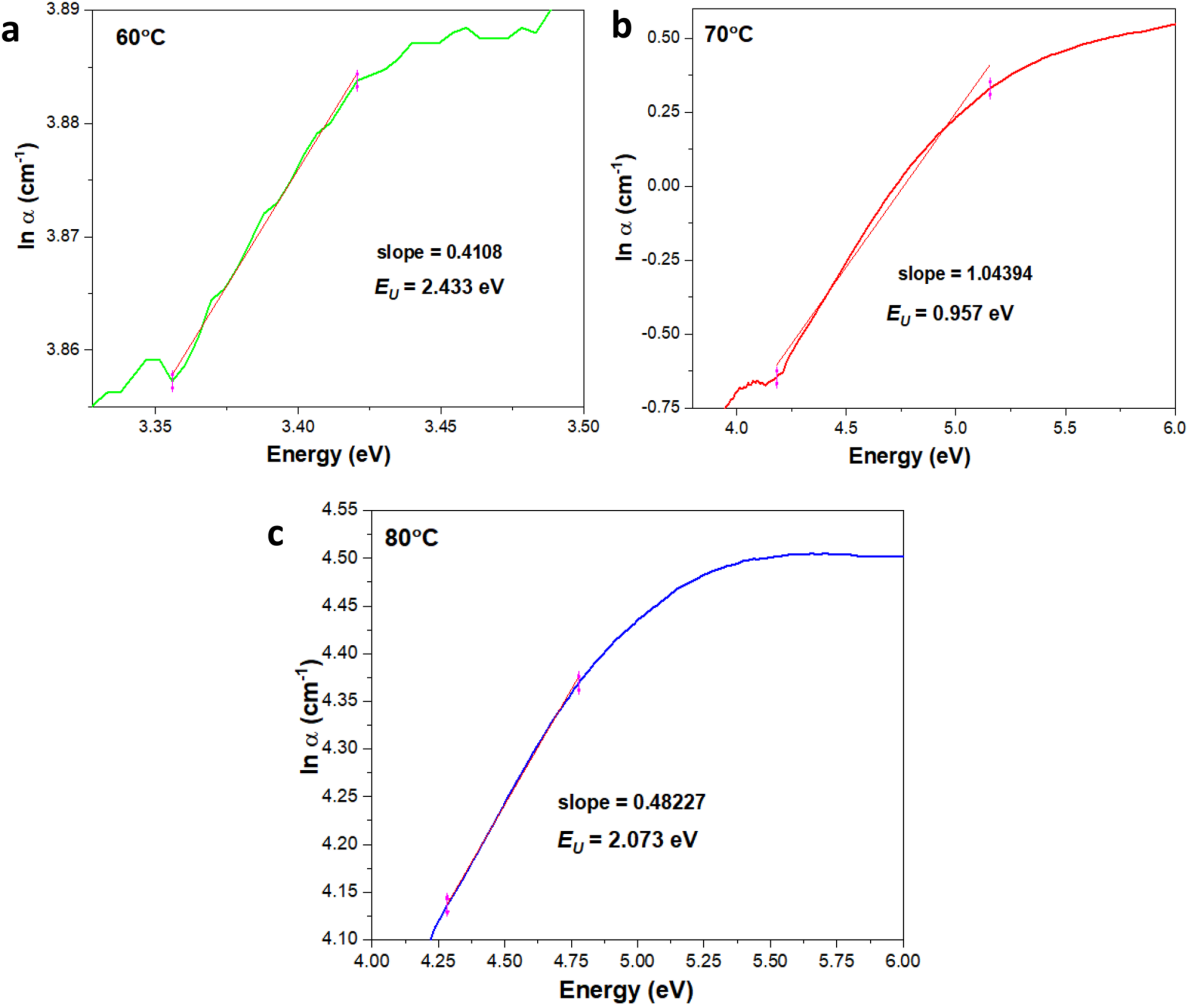
The absorption coefficient plots of Cu-based oxide nanostructures prepared at (**a**) 60, (**b**) 70 and (**c**) 80 °C. The curves are calculated using absorbance data with the best linear fitting model. The ln $\alpha$ scale yields the absorption edge of the Urbach region.

The different core-electron binding energy in the absorption coefficient data gives rise to the crystalline and structural/thermal disordered condition of the amorphous system. The absorption coefficient curve is close to the optical band edge in which the exponential part of an absorption tail (edge) represents the localised defect states extended to the binding energy of the core particles. Note that the exponential edge emerges when the sample has poor crystallinity, disordered structures and merely has amorphous characteristics. Principally, edge refers to the electronic density of states and controls the actual value of the energy gap via the alteration in the optical parameter (energy width or the localized state energy) of the Urbach tail, ${E}_{U}$. These parameters are known as Urbach empirical rules^94,95^, which give rise to the Urbach tail energy, ${E}_{U}$. The estimation of the ${E}_{U}$ for Cu-based oxide nanostructures are obtained from the reciprocal slope of the linear fitting from the plot of ln $\alpha$ (cm^−1^) versus photon energy (eV) as shown in Fig. 13. In the *ln* scale for $\alpha$, Urbach edge is shown to have a linear trend and the inverse of the slope represents the ${E}_{U}.$ The extension of the Urbach tail for all samples are approximately similar but with a reduction in the ${E}_{U}$ as the temperatures increased from 60 to 70 °C. The reduction of the ${E}_{U}$ from 2.433 to 0.957 and finally little increase to 2.073 eV at 80 °C evince that the effect of TA and temperatures are significant. Parallel to the microstrain values obtained from the W–H plot previously, the Urbach tailing mechanism indicates that at 80 °C, the TA allows more self-organisation structures and causes the increase in lattice strain (Table 4). In this respect, the smaller ${E}_{U}$ values is equal to the lesser energetic disorder (Urbach tail become narrower) in the present system. At some point, reducing the energetic disorder is favourable in decreasing the radiative loss below the bandgap. In any case, the oxide materials usually have less extended Urbach tail than the bare ones with the oxide clusters is much huger and yield a lower loss angle.

**Table 4:** Comparison of the crystallinity, Tauc bandgap, Urbach energies and W–H strains for all samples.

Sample	Crystallinity (%)	Bandgap (eV)		${\varepsilon }_{W{-}H}$
		*E*_*g*_	*E*_*U*_	
pH 7 (60 °C)	33.1	2.10 < E < 3.40	2.433	7.71468 $\times {10}^{-4}$
pH 7 (70 °C)	57.5	1.67 < E < 3.02	0.957	0.00266
pH 11 (80 °C)	38.6	2.93 < E < 3.87	2.073	0.00277

As for the comparison with existing literatures, the ${E}_{U }$ reported for CuO nanostructures is 1.01 eV, which is higher than that of other metal oxides^96^. Almost similar values of ${E}_{U}$ are observed when CuO is combined with other metal oxide systems such as in layered structures of metal oxides (e.g., SnO_2_–CuO–SnO_2_) giving the ${E}_{U}$ is in the range of 1.37 to 1.92 eV^97^. Also, the ${E}_{U}$ for CuO nanocomposites is far reduced as compared to the pure state of CuO nanostructures. For instance, the ${E}_{U}$ for the PMMA–PVA/CuO nanocomposite is between the range of 150 to 200 meV^98^. Polymer linkage or dopants change the overall condition by fundamentally reducing defects and impurities in the pure CuO nanosystem^99^. Additionally, the ${E}_{U}$ values are strongly affected by the substrate contribution in the system^100^. As for Cu_2_O nanoparticles, not many studies are reported on their Urbach energy. In most cases the small ${E}_{U}$ of CuO or Cu_2_O in the range of meV only can be found in the micrometer system but not in the nanoparticles ones because the crystalline quality at micrometer (μm) or higher scale nanometers are far ordered than the nanoparticles with the average particle size lower than 50 nm^100–102^. Generally, the significant surface area to volume ratio of the nanostructures itself cause the issue related to the inevitable surface defects. Hence, the degree of disorderliness within the matrices of the nanostructures is high^103^. In conclusion the ${E}_{U}$ quantify the system’s correlation on the static, disorder-induced localisation exponential-tail states and dynamic disorder from electron–phonon scattering as such the characteristics is essential in determining the photo-physic applications. Therefore, these types of as-synthesis Cu-based oxide nanostructures can be employed as one of the nanomaterials candidate specifically in the future development of organic photovoltaic semiconductor devices.

## Conclusion

In summary, the work shows that the Cu-based oxide nanostructures in the presence of tannic acid (TA) were successfully synthesised by a single-stage chemical reduction method. The as-produced CuO and Cu_2_O samples for each temperature show different pH values at different temperatures with varying copper to oxygen ratio. Data indicates that the pH change is mainly caused by the amount of quinone of TA produced during the chemical reaction and at 80 °C showed the highest pH obtained (pH 11). The influence of pH due to the alteration of quinone component amount on the formation of CuO and Cu_2_O in the produced sample elucidated the basis of pH-dependent competition kinetics between the reactions embark on either CuO or Cu_2_O formation. With the help of TA, the smallest mean crystallite size Cu-oxide based nanostructures produced is 23.6 nm at 80 °C and further confirmed by the TEM images. The results revealed that the amounts of semi-quinone and quinone of TA are the primary control factor in determining the nanostructures’ optical, morphologies, and structural properties. The extensive analysis of the formed nanostructures is described further.

The structural study disclosed the surface morphology, phase purity, elemental composition and diffraction microstrain. The structural errors are quite significant and can be seen from the FESEM micrograph. There is no linear trend in the crystallinity change with the change of the reaction temperature. The structural analysis involved the XRD data combined with the Williamson–Hall (W–H) analysis to understand the diffraction behaviour and presence of particle imperfections. The defects occur due to the combined reaction of heat and the alteration of the pH. This causes the strain in the crystal, where it usually manifests in the form of broadening and shape of the intensity peaks. The strain data by the W–H method is acceptable as the intercept and slope are positive, indicating the presence of microstrain and the morphology is not spherical in shape. The highest microstrain was found in the 80 °C sample.

Meanwhile, the Urbach energy obtained from the optical absorbance data inscribes the degree of atomic organisation of Cu-based oxide nanostructures caused by the TA gained from the optical interference. The energies are correlated well to the optical microstrain values obtained from W–H plots. The TA caused the samples to have low crystallinity due to the modification of surface structures and gradually increased the ${E}_{U}$ reading. The lowest ${E}_{U}$ obtained is at 70 °C with the highest crystallinity percentage. Furthermore, the comparative analysis carried out between first derivative absorbance data, ${E}_{U}$ and conventional Tauc plot revealed strong dependency on each other and immune to measurement-analysis details (i.e., improper spectra correction, formula, experimental error and different fitting range). The first derivative absorbance and ${E}_{U}$ are structure-sensitive properties and hence, suitable to investigate the nature of the disorder Cu-based oxide nanostructure produced from the present work.

Therefore, the current work has elucidated the structural and optical correlations for the as-synthesise Cu_2_O and CuO nanostructures. These results enable additional extensive investigations to validate further the correlation for these low crystalline oxide systems at a broader size range if possible. Also, at this stage, the reported outputs can provide relevant insight for their application in a self-power solar photovoltaic system.

## Data Availability

The raw/processed data required to reproduce these findings cannot be shared at this time as the data also forms part of an ongoing study. Further information on the data are available from the corresponding authors upon reasonable request.
